# Histological Study of Bone Marrow and Umbilical Cord Stromal Cell Transplantation in Regenerating Rat Peripheral Nerve

**DOI:** 10.22074/cellj.2016.3839

**Published:** 2016-01-17

**Authors:** Sam Zarbakhsh, Nasim Goudarzi, Maryam Shirmohammadi, Manouchehr Safari

**Affiliations:** 1 Research Center of Nervous System Stem Cells, Department of Anatomy, Faculty of Medicine, Semnan University of Medical Sciences, Semnan, Iran; 2 Department of Anatomy, Faculty of Medicine, Iran University of Medical Sciences, Tehran, Iran

**Keywords:** Bone Marrow Stromal Cells, Human Umbilical Cord Stromal Cells, Trans- plantation, Peripheral Nerve, Regeneration

## Abstract

**Objective:**

Bone marrow and umbilical cord stromal cells are multipotential stem cells that have the ability to produce growth factors that play an important role in survival and generation of axons. The goal of this study was to evaluate the effects of the two different mesenchymal stem cells on peripheral nerve regeneration.

**Materials and Methods:**

In this experimental study, a 10 mm segment of the left sciatic nerve of male Wistar rats (250-300 g) was removed with a silicone tube interposed into this nerve gap. Bone marrow stromal cells (BMSCs) and human umbilical cord stromal cells (HUCSCs) were respectively obtained from rat and human. The cells were sepa- rately cultured and transplanted into the nerve gap. The sciatic nerve regeneration was evaluated by immunohistochemistry, and light and electron microscopy. Moreover, histo- morphology of the gastrocnemius muscle was observed.

**Results:**

The nerve regeneration in the BMSCs and HUCSCs groups that had received the stem cells was significantly more favorable than the control group. In addition, the BM- SCs group was significantly more favorable than the HUCSCs group (P<0.05).

**Conclusion:**

The results of this study suggest that both homograft BMSCs and het- erograft HUCSCs may have the potential to regenerate peripheral nerve injury and transplantation of BMSCs may be more effective than HUCSCs in rat.

## Introduction

Peripheral nerve injury is a serious health problem for the society today affecting 2.8% of trauma patients with many of them acquiring life-long disability (1). Peripheral nerve injuries are traditionally treated with a nerve autograft that supplies structural support for sprouting axons originating from the proximal nerve stump. Major disadvantages of this method include: i. Multiple surgeries, ii. Loss of function or sensation at the donor site, iii. Need to sacrifice a healthy nerve and iv. Deficiency of graft material available for repair. Therefore, an effective alternative to the nerve autograft technique is required (2,4). One approach that has recently been noted is stem cell therapy which is likely to be effective for the treatment of neurotraumatic injuries and neurodegenerative diseases (5). Because stem cells are significant seeding cells for peripheral nerve regeneration, special consideration has been given to the development of a rich and accessible cellular storage of this cell-type (2,4). Bone marrow stromal cells (BMSCs) and human umbilical cord stromal cells (HUCSCs) are two types of MSCs that have the ability to differentiate into many cell lines such as fat, muscle, and neuron and Schwann cells (6,10). One of the greatest benefits of MSCs is that they are easily accessible and can be readily expanded in large-scale for transplantation (5). 

Moreover, BMSCs and HUCSCs are cells able to produce growth factors and anti-inflammatory cytokines that play important roles in survival and generation of axons. Some of these factors include nerve growth factor (NGF), brain-derived nerve growth factor (BDNF), vascular endothelial growth factor (VEGF), ciliary neurotrophic factor (CNTF) and glial-cell-line-derived growth factor (GDNF) (11,12). Thus, transplantation of BMSCs and HUCSCs may be useful for the regeneration of peripheral nerves after injury (11,15). 

In this study, we evaluated the effects of transplantation of BMSCs and HUCSCs on peripheral nerve regeneration. This was done to determine which cell-type is more effective based on the surviving factors of the stem cells. 

## Materials and Methods

### Animal model

In this experimental study, 24 male Wistar rats (250-300g) were obtained from Pasteur Institute of Iran. All animals had free access to food and water. Rats were randomly divided into 3 groups (n=8 in each group), namely the BMSC transplantation group, the HUCSC transplantation group and the control group. All procedures, including the use and care of animals, were approved by the Research Council of Iran University of Medical Sciences. 

### Bone marrow stromal cell culture

BMSC culture was prepared according to the method previously described by Zarbakhsh et al. (16). Briefly, after killing rats, femurs and tibias were dissected out. The bone marrow was ejected with 10 ml of Dulbecco’s Modified Eagle Medium (DMEM, Sigma, Aldrich) and cultured in DMEM containing 15% fetal bovine serum (FBS, Sigma Aldrich, USA), 2 mM L-glutamine (Sigma Aldrich, USA), and 100 mg/ml kanamycine (Sigma Aldrich, USA), incubated at 37˚C, with 95% humidity and 5% CO_2_. After 48 hours, nonadherent cells were removed by replacing the medium. The cells were expanded when they reached about 80% confluence and then passaged four times once every 7 days.

### Human umbilical cord stromal cell culture

Human umbilical cords of both sexes were collected from full-term births after either cesarean section or normal vaginal delivery with consent from the mothers according to the Institute’s Human Ethical Committee guidelines at Milad hospital, Tehran, Iran. The umbilical cord was washed in sterile phosphate buffered saline (PBS, Gibco, Germany) and blood vessels were removed. The remaining tissues were then cut into small pieces and were transferred into culture flasks with DMEM containing 10% FBS, 100 U/ml penicillin and 100 μg/ml streptomycin (Sigma Aldrich, USA), incubated at 37˚C with 95% humidity and 5% CO_2_. The non-adherent cells were washed with PBS after 48 hours and adherent cells were defined. HUCSCs were expanded when they reached about 80% confluence and then passaged three times once every 5 days (12,17,18). 

### Differentiation potential of the stem cells

To confirm the differentiation capacity of the stem cells, BMSCs and HUCSCs at passage 2 were individually treated with adipogenic induction medium for 21 days. Adipogenic induction medium comprised 10% FBS, 10 nM dexamethasone, 5 g/ml insulin, 0.5 M 3-isobutyl-1-methylxantine and 200 g/ml indomethacin in DMEM (Sigma Aldrich, USA). The cells were then fixed with buffered formalin (10%) in PBS for 10 minutes at room temperature and stained with oil-red O for 1 hour. Adipogenesis was observed after the differentiation process was completed (11,18,19). 

### Analysis of cell surface antigen markers

To analyze the expression of surface markers of the stem cells, flow cytometry was performed. At least 200,000 cells were incubated with fluorescencelabeled monoclonal antibodies against CD29-PE, CD34-FITC, CD44-PE and CD45-FITC (Sigma Aldrich, USA) Following a 10 minutes wash in PBS, the labeled cells were analyzed using a FACS Calibur flow cytometry apparatus (2,20). 

### Transplantation procedure

The transplantation procedure of the cells was performed according to that previously described by Zarbakhsh et al. (16). Briefly, rats were anesthetized and the left sciatic nerve was exposed at the mid-thigh. A 10 mm segment of the nerve was then removed and a 12 mm silicone tube was interposed into this nerve gap. Silicone tube was selected because it is the most reliable prosthetic in modeling the bridge conduit. Also, the inner diameter of silicone tube is similar to the rat sciatic nerve (21). Both ends of the nerve were fixed into the tube with a 10-0 nylon suture. The silicone tube in both experimental groups (BMSCs and HUSCs) was filled with fibrin gel seeded with about 500,000 cells. For the control group, it was filled with fibrin gel without any cells. Finally the skin was sutured with 5-0 silk. 

### Immunohistochemistry of the cells

BMSCs and HUCSCs were separately labeled with anti-BrdU (Bromodeoxyuridin, Sigma Aldrich, USA) and rhodamine (Sigma Aldrich, USA) as the primary and secondary antibodies respectively in the sciatic nerve to show the presence and also the viability of the transplanted cells after four weeks. The labeling protocol has been previously described (16,22,24). 

### Light microscopy

For light microscopy examination of the nerves, 12 weeks after transplantation, the rats were sacrificed and the regenerated nerves within the silicone tubes were harvested. The nerve grafts were fixed immediately in 2.5% glutaraldehyde solution. Then, the nerve tissues were post-fixed in 2% osmium tetroxide, dehydrated, embedded in Epon resin, semithin cross sectioned (700 nm) from the central portion using an ultra microtome and stained with toluidine blue. The nerve sections were observed under an Olympus light microscope (PROVIS Ax70, Japan). The number of axons and blood vessels, and the diameter of the axons were calculated in randomly selected fields (2,11,21,25,26). 

### Electron microscopy

With preparation of specimens for toluidine blue staining, the thickness of the myelin sheath was also evaluated using transmission electron microscopy (TEM). Ultrathin sections (70 nm) were placed on 300-mesh copper grids, stained with 5% uranyl acetate in 70% ethanol (Razi, Iran) for 5 minutes, dried, counterstained with lead citrate for 5 minutes, dried and examined on a Zeiss EM 10 CR TEM (Jenna, Germany) (2,11,21,27). 

### Histomorphology of the muscle

Along with the removal of the left sciatic nerve, the left gastrocnemius muscle was also removed. Cross sections of the muscle were fixed in phosphate buffered saline containing 4% formaldehyde, and routine paraffin-embedded sections were produced. The 5 µm paraffin-embedded sections were rehydrated using xylene and a graded alcohol series, and stained with hematoxylin and eosin (H&E) to observe the cellular morphology, and fibrosis and degenerative changes under a light microscopy (Olympus, Japan) (21). 

### Statistical analysis

All data were analyzed by one-way ANOVA followed by the Tukey test. Obtained data were presented as mean ± SD. The significance level of P<0.05 was considered statistically significant. 

### Results

#### Culture, differentiation and characterization of bone marrow stromal cells and human umbilical cord stromal cells 

**Fig.1:**
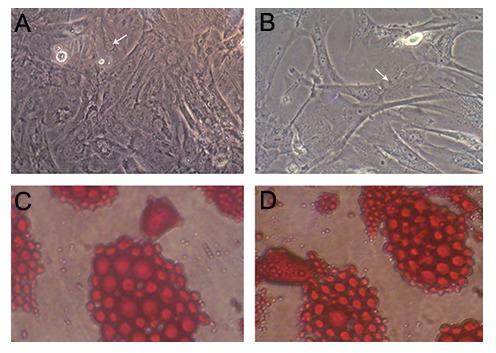
Cultured A. Bone marrow stromal cells (BMSCs), B. Human umbilical cord stromal cells (HUCSCs) at passage 3, C. Differentiation potential of bone marrow stromal cells and D. Human umbilical cord stromal cells [into adipocytes as red drops (Oil-red O staining)] (×200). Arrows show the nucleus of the cells.

BMSCs are typically isolated from other cells by sticking to plastic. Nonadherent cells were removed by changing the medium at 72 hours and every 3 days thereafter. By day 7 in culture, the attached cells had developed into an adherent layer containing abundant dispersed spindle-like cells. By day 14 in culture, the primary BMSCs had proliferated and started to form a nearly continuous layer comprised mainly of fibroblast-like cells. By repeating passages, the fibroblast-like cells became morphologically homogeneous (Fig .1A). 

The isolated HUCSCs began to attach to the floor of flask after 6 hours. At 24 hours, the attached cells exhibited ovoid and spindle-shaped fibroblast-like morphology. The HUCSCs proliferated markedly faster than the BMSCs and in the third passage became relatively homogeneous with elongated spindle-like morphology (Fig .1B). 

To determine the differentiation potential of the stem cells, BMSCs and HUCSCs were treated with adipogenic induction medium and adipogenesis was seen with oil-red O staining (Fig .1C,D). 

**Fig.2:**
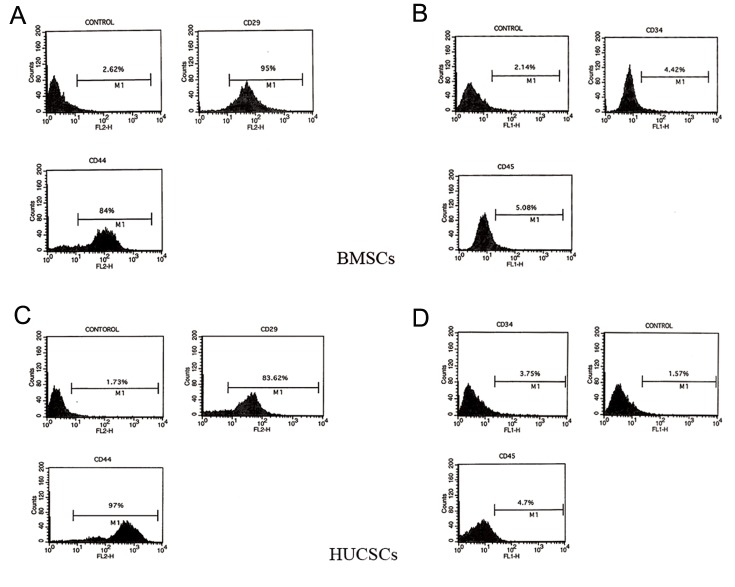
Flow cytometry results showing bone marrow stromal cells (BMSCs) and human umbilical cord stromal cells (HUCSCs) being positive for A, C. CD29 and CD44 and B, D. Negative for CD34 and CD45.

Flow cytometry analysis demonstrated that these stem cells were immunopositive for markers of mesenchymal stromal stem cells, CD29 and CD44, and were immunonegative for hematopoietic markers, CD34 and CD45 (Fig .2). 

### Immunohistochemical analysis of cells

**Fig.3:**
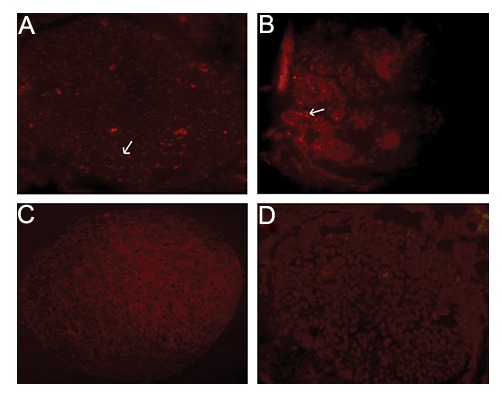
Immunohistochemistry of A. Bone marrow stromal cells (BMSCs), B. Human umbilical cord stromal cells (HUCSCs), C. Labeled with anti-BrdU antibody and negative control [without the primary antibody of BMSCs and D. HUCSCs] in cross sections of the sciatic nerves 4 weeks after transplantation (×100). Arrows show the labeled cells.

The BMSCs and the HUCSCs were successfully labeled as red spots were visible in the cross sections of the sciatic nerves. These results confirmed the presence and also the viability of the transplanted cells into the silicon tube between the two ends of the nerve (4 weeks after transplantation) (Fig .3). 

### Histological comparisons of the nerve

**Fig.4:**
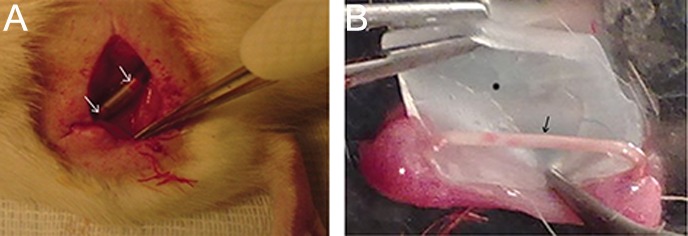
A. The silicone tube between the ends of the nerve after surgery. Arrows show the two ends of the nerve in the silicone tube and B. Arrow shows regenerated nerve in the silicone tube 12 weeks after transplantation. The black star shows the opened silicone tube and the white star shows connective tissue around the nerve and the silicone tube.

Twelve weeks after transplantation, the surface of the tube was replaced with vascularized connective tissue. In all groups, axons traversed the whole length of the silicone tube. In the silicone tube, there was a matrix consisting of capillaries, regenerated axons and connective tissue. There was almost no inflammatory reaction within the tube (Fig .4). 

**Fig.5:**
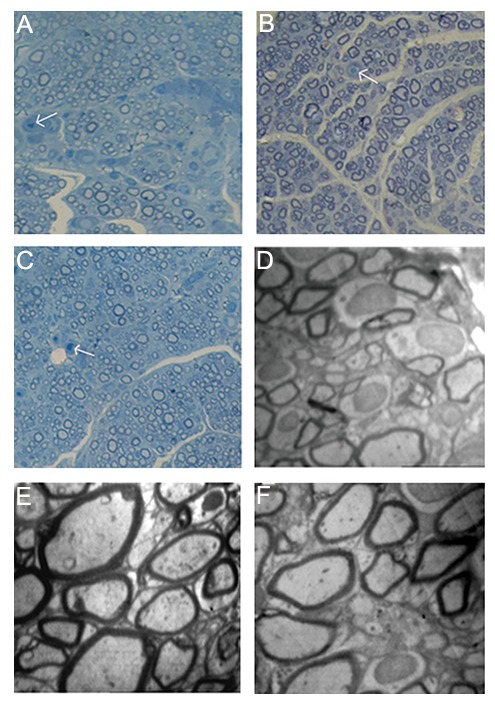
A-C. Semithin cross-sections stained with Toluidine Blue (×400) and D-F. Transmission electron photomicrographs of cross sections of the regenerated sciatic nerves 12 weeks after transplantation. A, D. The control group showed a relatively low number of axons and small myelinated axons with thin myelin sheaths (magnification D: ×1500), B, E. The bone marrow stromal cell group showed regeneration with myelinated axons and proper myelin sheaths (magnification E: ×2000) and C, F. The human umbilical cord stromal cell group showed a fairly good number of axons and many myelinated and non-myelinated axons with preserved myelin sheaths (magnification F: ×2000). Arrows show the blood vessels.

**Fig.6:**
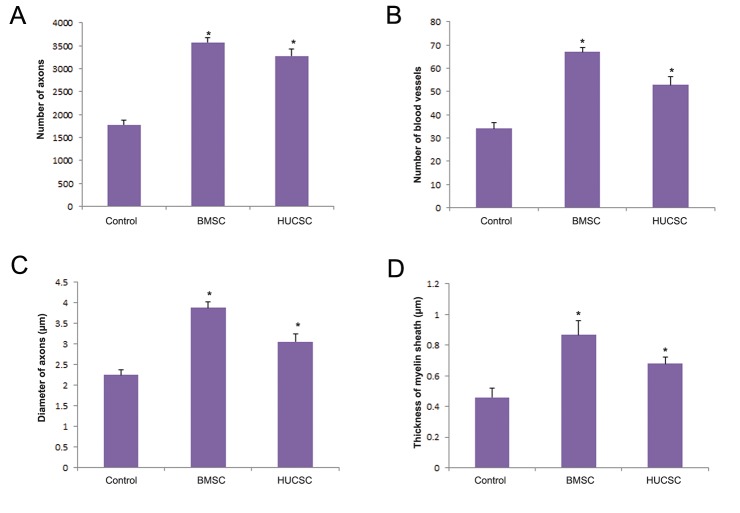
Histological differences of the sciatic nerve between the control and the experimental groups [bone marrow stromal cells (BMSCs) and human umbilical cord stromal cells (HUCSCs)] 12 weeks after transplantation. A. The number of axons, B. The number of blood vessels, C. The diameter of axons and D. The thickness of myelin sheath. *; P<0.05 vs. control group.

Histological evaluation of the sciatic nerve regeneration was undertaken using light and electron microscopy. The results of light microscopy consisted of the number of axons and blood vessels, and the diameter of axons (Fig .5A-C). The results of electron microscopy consisted of the thickness of the myelin sheath (Fig .5D-F). In the experimental groups, a typical pattern of nerve regeneration was observed consisting of a newly formed perineurium that included nerve fascicles of myelinated and unmyelinated axons with further neovascularization. However, smaller axons with thinner myelin sheath and less neovascularization were seen in the control group. All histological data showed that the regeneration in the experimental groups (BMSCs and HUCSCs) was significantly greater than the control group. Similarly, the regeneration in the BMSC group was significantly greater than the HUCSC group (P<0.05, Fig .6). 

### Histomorphological observation of the muscle 

**Fig.7:**
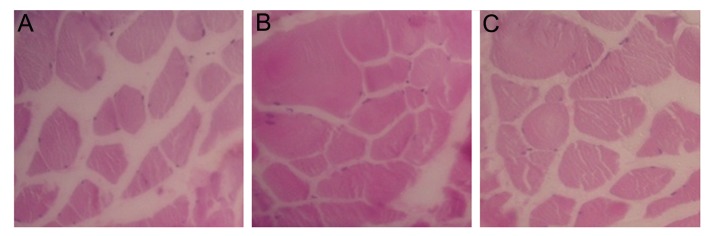
Cross sections of the gastrocnemius muscles (12 weeks after transplantation) stained with Hematoxylin and Eosin (H&E) (×200). A. The control group showed severe muscle atrophy with increased fibrosis, B. The bone marrow stromal cell (BMSCs) group showed aligned muscle bundles with low fibrosis and C. The human umbilical cord stromal cell (HUCSCs) group displayed mild fibrosis.

Twelve weeks after transplantation, histomorphological observation of the gastrocnemius muscle showed there were closely packed muscle fibers in the experimental groups (BMSCs and HUCSCs) while generalized muscle atrophy with increased fibrosis was observed in the control group (Fig .7). 

## Discussion

Nerve autograft is common used for peripheral nerve defects but the length of available nerve grafts is limited. Nerve regeneration using nonbiological materials such as silicone tubes has been attempted for large nerve defects that cannot be treated by nerve autografting (4,26). Recent experimental studies demonstrate the beneficial effects of transplanted MSCs in regeneration of damaged peripheral nerve tissues (6,9,11,28). We therefore aimed to evaluate the effects of transplantation of BMSCs and HUCSCs on histological regeneration of peripheral nerve. 

BMSCs and HUCSCs as two types of MSCs that, according to several reports, can repair peripheral nerve injuries (6,9,11,28). Comparison of different cell types may result in the identification of cells with a greater ability to regenerate peripheral nerves. This may lead to a novel clinical approach for applying these cells to replace the highly invasive peripheral nerve autograft technique (2,4). 

We show that BMSC and HUCSC cells could survive in the silicone tube within the gap between the two ends of the nerve after 4 weeks of transplantation. These findings were consistent with various reports (2,16,24,29,30). We also show that these stem cells have the ability to differentiate into another cell type (adipocyte) which agrees with other reports(9,18,20,28,31,32). 

Moreover, based on surface marker expressions, BMSCs and HUCSCs had the characteristics of multipotent MSCs, also consistent with other reports (2,17,20). 

MSCs are emerging as strong candidates particularly for cellular therapies for at least three reasons. First, they can be isolated from a wide range of autologous sources with some readily accessible. Secondly, their high proliferative potential allows for rapid expansion *ex vivo*, while maintaining multipotentiality. Finally, these cells produce nerve growth factors such as NGF, BDNF, GDNF, CNTF, VEGF and NT-3 as well as substantial extracellular matrix proteins such as collagen I, collagen IV, fibronectin and laminin (11,15,33,35). 

These results were consistent with several reports (6,9,36,38). The greater regeneration of the number, diameter and myelin thickness of the axons in the experimental groups as compared with the control group could probably be due to the role of the stem cells in producing growth factors stated above and enabling cells to differentiate into Schwann cells that directly support the growth of axons (11,15,33,35,38). Fan et al. (13) have shown that VEGF can cause neovascularization. Therefore, the greater number of blood vessels in the experimental groups as compared with the control group may be due to the role of VEGF that was produced by the stem cells. The greater regeneration in the BMSC group than the HUCSC group is probably due to homografts being often more favorable than heterografts (39). In recent studies, heterograft from human into experimental animals has been performed extensively. Zhilai et al. (12) transplanted HUCSCs into rat with spinal cord injury. Zhang et al. (17) transplanted HUCSCs into ataxic mice. Moradi et al. (40) cultured Schwann cells from aborted human fetuses and transplanted the cells into rats with spinal cord injury. Lee et al. (30) transplanted HUCSCs into dogs with spinal injury. Gartner et al. (41) evaluated the effects of HUCSCs on rat sciatic nerve regeneration after neurotmesis injuries. Due to the difficulties in providing homograft cells for humans, heterograft cells may provide a bright future for cell therapy. 

The risk of rejection in our study was minimal since the MSCs secrete immunomoulatory factors and anti-inflammatory cytokines, thus modulating immune and inflammatory responses (12,41,44). 

Since both cell types (BMSCs and HUCSCs) have the ability to release neurotrophic factors and extracellular matrix proteins, comparative analysis may lead to a more efficient treatment approach based on the measure of peripheral nerve regeneration. 

## Conclusion

The results of this study suggest that both homograft BMSCs and heterograft HUCSCs may have the potential to regenerate peripheral nerve injury with BMSC transplantation being more effective than HUCSC in rat. 
